# Withanolide derivatives: natural compounds with anticancer potential offer low toxicity to fertility and ovarian follicles in mice

**DOI:** 10.1590/1984-3143-AR2024-0027

**Published:** 2024-10-21

**Authors:** Gaby Judith Quispe Palomino, Homero Ygnacio Celiz, Francisco Denilson Rodrigues Gomes, Gildas Mbemya Tetaping, Marco Aurélio Schiavo Novaes, Késya Amanda Dantas Rocha, Ramon da Silva Raposo, Rebeca Magalhães Pedrosa Rocha, Ana Beatriz Graça Duarte, Otilia Deusdênia Loiola Pessoa, José Ricardo Figueiredo, Naiza Arcângela Ribeiro de Sá, Ana Paula Ribeiro Rodrigues

**Affiliations:** 1 Laboratório de Manipulação de Oócitos e Folículos Ovarianos Pré-antrais – LAMOFOPA, Faculdade de Medicina Veterinária, Universidade Estadual do Ceará, Fortaleza, CE, Brasil; 2 Universidade da Integração Internacional da Lusofonia Afro-Brasileira, Redenção, CE, Brasil; 3 Laboratório de Análise Fitoquímica de Plantas Medicinais, Universidade Federal do Ceará, Fortaleza, CE, Brasil; 4 Centro de Biologia Experimental, Universidade de Fortaleza, Fortaleza, CE, Brasil; 5 Departamento de Morfologia, Faculdade de Medicina, Universidade Federal do Ceará, Fortaleza, CE, Brasil

**Keywords:** chemotherapy, cytotoxicity, reproductive function, apoptosis, infertility

## Abstract

Anticancer therapy often leads to premature ovarian insufficiency (POI) and infertility due to the extreme sensitivity of the ovarian follicle reserve to the effects of chemotherapy. Withanolides are known for their cytotoxic effect on cancer cells and low cytotoxicity on non-malignant or healthy cells. Therefore, this study aimed to investigate the *in vivo* effects of three withanolides derivatives: 27-dehydroxy-24,25-epoxywithaferin A (WT1), 27-dehydroxywithaferin A (WT2), and withaferin A (WTA) on fertility, and the ovarian preantral follicles of young female mice. To achieve this, mice received 7 intraperitoneal doses of WT1, WT2, or WTA at a concentration of 2 mg/kg (Experiment I) and 5 or 10 mg/kg (Experiment II) over 15 alternate days. *In experiment I*, two days after administration of the last dose, half of the mice were mated to evaluate the effects of withanolides on fertility. The other half of the mice, as well as all mice from *experiment II*, were sacrificed for histological, inflammation, senescence, and immunohistochemical analyses of the follicles present in the ovary. Regardless of the administered withanolide, the concentration of 2 mg/kg did not show toxicity on the follicular morphology, ovarian function, or fertility of the mice. However, at concentrations of 5 and 10 mg/kg, the three derivatives (WT1, WT2, and WTA) increased follicular activation, cell proliferation, and ovarian senescence without affecting inflammatory cells. Furthermore, at a concentration of 10 mg/kg, the three withanolides showed intensified toxic effects, leading to DNA damage as evidenced by the labeling of γH2AX, activated Caspase 3, and TUNEL. We conclude that the cytotoxic effect of the tested withanolide derivatives (WT1, WT2, and WTA) in the concentration of 2 mg/kg did not show toxicity on the ovary. However, in higher concentrations, such as 10 mg/kg, toxic effects are potentiated, causing DNA damage.

## Introduction

According to several reports in the literature, advances in cancer treatment over the last decade have increased survival rates for both adult and pediatric patients (Siegel et al., 2024). Unfortunately, due to the gonadotoxic effects of chemotherapy drugs, female infertility is reported to be a major concern among women of reproductive age diagnosed with cancer (Letourneau et al., 2012; Lichota and Gwozdzinski, 2018; Atteeq, 2022). Several authors (Spears et al., 2019; Chon et al., 2021; Sellami et al., 2023; Hao et al., 2019; Kashi and Meirow, 2023; Kashi et al., 2023) have shown that anticancer therapies could cause accelerated loss of ovarian follicular reserve, leading to the condition known as premature ovarian failure (POF). Therefore, many efforts have been made to identify drugs or compounds with chemotherapeutic potential and low adverse effects on the population of ovarian follicles, especially preantral follicles.

In this context, withanolides, a class of secondary metabolites extracted from different plants, have been the subject of great interest not only because of their anti-inflammatory (Abdeljebbar et al., 2009), anxiolytic (Bhattacharya et al., 2000), cytotoxic (Paul et al., 2021), but especially anticancer (Zhang et al., 2023; WalyEldeen et al., 2023) properties, both *in vivo* and *in vitro* (Kumar et al., 2010; Guerreiro et al., 2019; Zhao et al., 2021). Withanolide derivatives trigger apoptosis in cancer cells by activating both the intrinsic and extrinsic pathways of programmed cell death (Samadi, 2015). In an *in vitro* study carried out by our team, we demonstrated the cytotoxicity of withanolide D, extracted from *Acnistus arborescens*, on the population of goat preantral follicles, similar to that exerted by paclitaxel (Guerreiro et al., 2019), a chemotherapy drug widely used for the treatment of ovarian cancer (Kampan et al., 2015; Sati et al., 2024). In a second study also carried out *in vitro* with preantral follicles included (primordial, primary, and secondary) or isolated from the ovary (secondary follicles) of the same species, withanolide D associated with melatonin also showed follicular toxicity (Palomino et al., 2021).

However, derivatives of withanolides, known to exhibit low cytotoxicity towards non-malignant or healthy cells (Zhang et al., 2011; Nagy et al., 2020), such as withaferin A (WTA), also demonstrate activity against cancer cell lines. Among these, melanoma cells (B16F10 and SKMEL28) and breast cancer (Hs578T) can be mentioned, as reported by Zhang et al. (2011). Liver (Nagy et al., 2020) and ovarian cancer cells could be also mentioned, both in *in vivo* (Kakar et al., 2017; Straughn and Kakar, 2019) and *in vitro* studies (Fong et al., 2012; Kakar et al., 2012). Recently, some withanolides were isolated from the plant *Athenaea velutina* with anti-inflammatory and antiviral activities (Dantas Rocha et al., 2022; Alves et al., 2023). Additionally, others withanolides, including WT1, WT2 and WTA were evaluated for their cytotoxic activity, although the data have not yet been published, WT1, WT2 and WTA showed *in vitro* cytotoxicity in different cell lines: HL-60 (leukemia), HCT-116 (colon), PC-3 (prostate), SNB-19 (glioblastoma) and non-tumorous murine fibroblasts (data provided by Dr. Pessoa, from the Laboratory of Phytochemical Analysis of Medicinal Plants of Federal University of Ceará, Brazil). Thus, these significant reports have prompted the need to concurrently pursue the investigation of these derivatives on ovarian follicles, which were previously unexplored.

Therefore, this study aimed to evaluate the *in vivo* effects of withanolide derivatives (WTA, WT1, and WT2) on fertility and preantral ovarian follicles (follicular reserve) of young female mice. The parameters evaluated included follicular morphology, signs of inflammation and cellular senescence, immunostaining for FOXO3a, Ki67, γH2AX, activated Caspase 3, and DNA fragmentation proteins (TUNEL).

## Methods

### Ethics approval

All experimental protocols were approved by the Animal Care and Use Committee (ACUC) from UNIFOR (N° 5418220621) and according to international guidelines for animal care.

### Animals and chemicals

C57BL6J female mice (50-60 days of age; weight 20-25 g) were obtained from the experimental biology core of the University of Fortaleza (UNIFOR). Experimental mice were housed in polyethylene boxes with free access to a standard chow diet and water and subjected to 12-hour light-dark cycles. All efforts were made to minimize the number of animals while ensuring statistically significant data.

Unless indicated otherwise, the culture medium and the other chemicals used in this study were purchased from Sigma Chemical Co. (St. Louis, MO, USA). WT1, WT2, and WTA at a purity of > 95% were kindly provided by the Laboratory of Phytochemical Analysis of Medicinal Plants II, from the Federal University of Ceará.

### Experimental design

To investigate the *in vivo* effects caused by the drugs WT1, WT2 and WTA on the survival and development of preantral ovarian follicles and the fertility of young mice, this study was conducted in two experiments, as described below:

#### Experiment I - treatment with 2 mg/Kg of WT1, WT2 or WTA

Forty female mice were randomly distributed into 4 treatments (n = 10 per treatment) to receive 7 intraperitoneal (I.P.) doses of 2 mg/kg of WT1, WT2, WTA, or saline solution (control; CTR) for 15 alternate days. Seventeen days after the administration of the first dose, half of the females (n = 5) from each treatment were euthanized by an overdose of ketamine/xylazine solution followed by cervical dislocation. Immediately afterward, ovarian pairs from each experimental group were harvested, and the surrounding tissues were dissected using 26-gauge (26 G) needles attached to 1 mL syringes, under a stereomicroscope (SMZ 645 Nikon, Tokyo, Japan) and fixed for analysis of follicular morphology, as well as the presence of inflammatory and senescent cells, respectively to identify inflammation and ovarian senescence.

To evaluate the effect of the drugs on fertility, the remaining 20 females were distributed into groups of 2 to 3 mice per cage and housed with a single male C57BL/6J mouse (aged 12 weeks to 1 year) for 15 consecutive days for mating. This protocol corresponds to three consecutive reproductive cycles and, consequently, three ovulations (Calanni-Pileri et al., 2022). At the end of this period, the males were removed, and pregnancy was confirmed by visual inspection or palpation of the abdomen to detect fetuses. The non-pregnant females were mated with the males once again, following the same system.

#### Experiment II - treatment with 5 or 10 mg/Kg of WT1, WT2 or WTA

Among the female offspring born from the mating conducted in experiment I, 35 (n = 5 per treatment) received I.P. injections of saline solution (control; CTR), and the drugs WT1, WT2, or WTA at doses of 5 or 10 mg/Kg. To avoid synergistic or antagonistic effects resulting from the combination of drugs, the females distributed in each treatment in Experiment II were the offspring of mothers from the same groups as those in Experiment I. The protocol for administering the drugs and sacrificing the animals was similar to that adopted in Experiment I. At the end of the experiment, the ovaries were collected and destined for the same analyses conducted in Experiment I, including immunolocalization of proteins (FOXO3a, Ki67, γH2AX, activated Caspase 3) and DNA fragmentation (TUNEL).

### Histological analysis

The ovaries of animals from experiments I and II were fixed in Davidson's solution for 12 hours at room temperature, then dehydrated using graded ethanol, clarified in xylene, and embedded in paraffin. The paraffin-embedded tissue blocks were serially sectioned at a thickness of 5 µm, and sections were stained with periodic acid–Schiff (PAS) to facilitate morphological examination of ovarian follicles using a Nikon microscope (Tokyo, Japan). Follicles were classified as primordial (one layer of flattened or flattened and cuboidal granulosa cells), primary (a complete layer of cuboidal granulosa cells surrounding the oocyte), and secondary (two or more layers of cuboidal granulosa cells and no sign of antrum formation) follicles (Palomino et al., 2021). Furthermore, the follicles were classified as histologically normal if no overt signs of degeneration were noted, which included a retracted or vacuolated oocyte, condensed nuclear chromatin, disorganized granulosa cells detached from the basement membrane, and/or cell swelling. To evaluate follicular activation, only morphologically normal follicles were recorded, and the proportion of primordial and growing (primary and secondary) follicles was calculated in different treatments. To avoid double counting of the same follicles, follicles were counted on the first section of the entire ovary in which the centrally located nucleus of the oocyte appeared. All counts were performed blindly.

### Assessment of inflammatory cells

The presence of inflammatory cells was observed only in antral follicles examined on PAS-stained slides under a light microscope (Nikon, Japan). This type of cell was not found in preantral follicles. Under a 40x objective lens, the whole ovarian area was evaluated for the presence of inflammatory cell aggregates. We then examined the level of infiltration in the antral follicles under a 100x objective lens. For quantification of inflammatory cells, 4 fields of 10 different antral follicles were obtained (40 fields per animal in each treatment). The inflammatory infiltrate was quantified by area. The data were presented as the number of inflammatory cells by area in the antral follicles.

### Sudan black B staining and lipofuscin analysis

For histological analysis of lipofuscin granules, ovarian tissue fragments included in paraffin blocks were sectioned at a thickness of 5 µm. The modified protocol described by Lima et al. (2023) was used. Briefly, the slides were deparaffinized with xylene, washed in a gradient of alcohols, stained with Sudan Black B (SBB) for 2 min, immersed in 50% ethyl alcohol for 2 min, subsequently in distilled water for 2 min, and finally mounted with 40% glycerol. After the procedure, the slides were evaluated for labeling the lipofuscin granules under an optical microscope. Four images per animal were obtained in 40× objectives, and cells with positive staining for lipofuscin were quantified. The number of pixels in the images was determined using Image J software, and subsequently, the average number of labeled cells was calculated by dividing the number of labeled cells by the total area of the evaluated image.

### Immunohistochemistry (IHC)

For immunolocalization of the FOXO3a protein, antigen retrieval was performed by incubating slides in EnVision FLEX Target Retrieval Solution High pH (K8005, Dako, Santa Clara, CA, USA) at 95–100 °C for 5 minutes in a pressure cooker. endogenous peroxidase blockade was achieved through two successive 15 min washes using 10% H_2_O_2_ in methanol (0.01 M, pH 6), interspersed with washes in EnVision FLEX Wash Buffer (K8024, Dako). Then, incubation was carried out for 30 min at room temperature with anti-FOXO3a rabbit polyclonal primary antibody (diluted 1:80 - ab70315; Abcam Inc., Cambridge, MA, USA). Subsequently, following the repetition of the peroxidase blockade step, slides were incubated for 30 minutes with goat anti-rabbit IgG secondary antibody (1:200). The slides were incubated for 30 min with avidin-biotin enzyme complex (ABC; Vector laboratories, 465 Burlingame, CA, USA) for reaction with 3,3′-diaminobenzidine in chromogen solution (DAB) for 5 min. Hematoxylin and a 0.5% ammonia solution were used for counterstaining. The negative control was conducted by omitting the primary antibody. Preantral follicles with cytoplasmic labeling (brown staining) for FOXO3a were considered active (Uhlenhaut and Treier, 2011). The images were analyzed for color intensity, measured in pixels, using ImageJ software.

### Immunofluorescence (IF)

The immunolocalization of Ki67, γH2AX, and active Caspase 3 was performed by IF. Subsequently, antigen retrieval was performed by incubating the slides containing sections of ovarian tissue and secondary follicles in 0.01 M sodium citrate buffer (pH 6) at 95–100 °C for 5 minutes in a pressure cooker. Then, slides were incubated in PBS solution containing 1% (w/v) BSA for unspecific blockade for 1 hour at room temperature. Then, slides were incubated overnight at 4 °C with rabbit polyclonal Ki67 (1:500 - ab15580; Abcam Inc., Cambridge, MA, EUA), mouse monoclonal γH2AX (phospho S139) (1:200 ab26350, Inc. Abcam, Cambridge, MA) or rabbit polyclonal active anti-Caspase 3 (1:1000 ab4051, Abcam Inc., Cambridge, MA) primary antibody. After incubation with the primary antibody, slides were washed twice in 1% PBS, and incubated with donkey polyclonal to rabbit IgG (1:500; ab150113, Abcam Inc., Cambridge, MA) for Ki67 and active Caspase 3 and goat polyclonal to mouse IgG (1:200; ab150113, Abcam Inc., Cambridge, MA) secondary antibodies conjugated to Alexa Fluor® 488 for γH2AX by 1 hour at room temperature. Slides were mounted with Fluoroshield Mounting Medium with DAPI (Abcam Inc., Cambridge, MA, USA). Immunostaining was assessed using a confocal laser-scanning microscope (LSM 710, Zeiss, Oberkochen, Germany). The negative control was established by omitting primary antibodies. Preantral follicles were regarded as positive for the proteins if they emitted green fluorescence (Guerreiro et al., 2019). Subsequently, the images were analyzed for color intensity, expressed in pixels, using ImageJ software.

### DNA fragmentation assay

DNA fragmentation was analyzed by TUNEL (Terminal deoxynucleotidyl transferase-mediated biotinylated deoxyuridine triphosphates nick end-labeling), using the In Situ Cell Death Detection Kit, POD (Roche Applied Science, Mannheim, BW, Germany), according to the manufacturer's instructions. The ovaries were initially fixed in 4% paraformaldehyde in PBS (pH 7.2), then dehydrated and embedded in paraffin wax. Tissue sections (5 µm) mounted on Superfrost Plus slides (Knittel Glass, Bielefeld, NW, Germany) were deparaffinized with Citrisolve (Fisher Scientific, Ottawa, Ontario, Canada) and subsequently rehydrated in a graded series of ethanol. Antigen retrieval was carried out by incubating the tissue sections in 0.01 M sodium citrate buffer (pH 6.0) for 5 minutes in a pressure cooker. To block endogenous peroxidase, slides were incubated with 3% H_2_O_2_ in methanol for 1 hour at room temperature. After washing, the slides were incubated with the TUNEL mix for 1 hour at 37°C. Converter POD was added, and the localization of protein expression was demonstrated by incubation with DAB (0.05% DAB in Tris/HCl, pH 7.6, 0.03% H_2_O_2_). Finally, the sections were counterstained with hematoxylin. Follicles were identified as having fragmented DNA when oocytes with dark brown stained nuclei were detected (Santos et al., 2014). As an internal positive control, sections were treated with 10 U/mL DNase I (InvitrogenTM, Carlsbad, CA) for 15 min at RT before incubation with the TUNEL reaction mixture to induce nonspecific breaks in the DNA. The negative control sections omitted the terminal deoxynucleotidyl transferase enzyme (Tarumi et al., 2009). Following this, the images were analyzed for color intensity, expressed in pixels, using ImageJ software.

### Statistical analysis

Statistical analysis was performed out using the SPSS software (Version 23.0, SPSS, Inc., Chicago, IL, USA). The morphological evaluation, quantity of inflammatory cells, and FOXO3a immunostaining exhibited parametric behavior, and the comparison between the drugs (WT1, WT2, or WTA) with the same concentration was assessed using the ANOVA test. Meanwhile, the comparison between concentrations of 5 and 10 mg/kg within the same drug was analyzed using the t-student test. All other parameters evaluated exhibited non-parametric behavior and were assessed using the Kruskal-Wallis H test and Mann-Whitney U test. Significance was determined when P < 0.05.

## Results

### Evaluation of ovarian and reproductive function indicators after administration of WTI, WT2, and WTA

#### Fertility

Mice from all treatments (CTR, 2 mg/Kg WT1, WT2, or WTA) became pregnant and showed no differences (P > 0.05) in the rate of live-born offspring (~ 7 pups/female).

#### Follicular morphology

**Figure 1:**
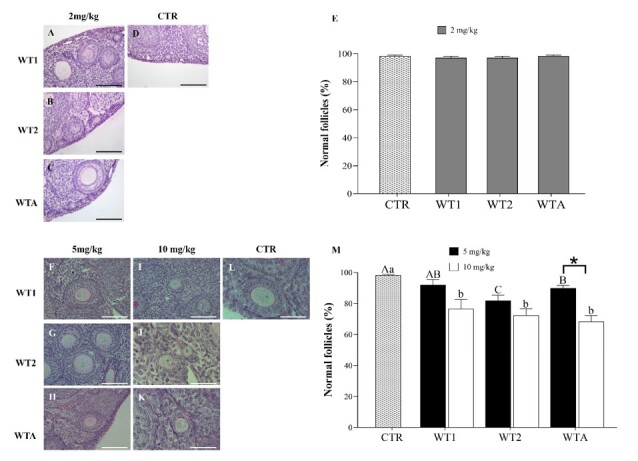
Micrographs and percentage (± s.e.m.) of morphologically normal follicles in ovarian tissue of female mice given doses of 2 mg/kg (A-D), 5, and 10 mg/kg (F-L) of WT1, WT2, and WTA. Percentage (± s.e.m.) of morphologically normal follicles in ovarian tissue of female mice given doses of 2 (E), 5 and 10 mg/kg (M) of WT1, WT2, and WTA. ^A,B,C^indicate differences in the comparison among CTR and 5 mg/kg of WT1, WT2, and WTA; ^a,b^indicate differences among CTR and 10 mg/kg of WT1, WT2, and WTA. *Indicates differences between 5 and 10 mg/kg of the same withanolide. Scale bar = 50 μm.

A total of 1,250 and 2,000 preantral follicles (primordial, primary, and secondary) were analyzed in experiments I and II, respectively. Representative images of the follicles are shown in Figures 1A-D and 1F. As shown in Figure 1E, in experiment I, follicle morphology did not differ among groups (P > 0.05). In experiment II, the concentration of 5 mg/Kg reduced (P < 0.05) the percentage of morphologically normal follicles in the WT2 and WTA groups compared to the CTR (Figure 1G), while at the concentration of 10 mg/Kg, this effect was observed in all withanolide-treated groups (WT1, WT2, and WTA). Furthermore, in the presence of WTA, the concentration of 10 mg/Kg was more toxic than the 5 mg/Kg concentration, significantly reducing (P < 0.05) the total percentage of normal follicles.

#### Follicular activation by analyses of morphology and FOXO3a expression

**Figure 2:**
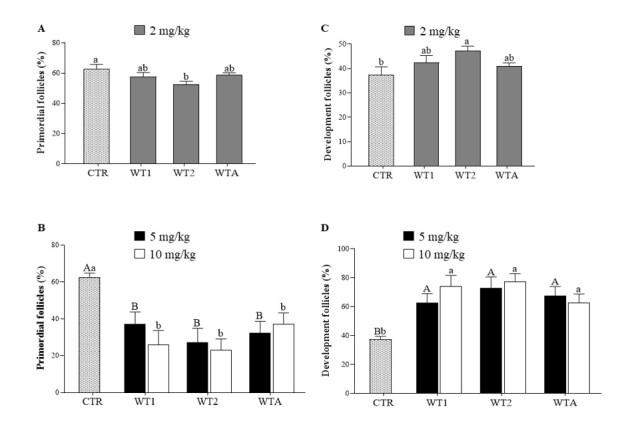
Percentage (± s.e.m.) of primordial (A and B) and developing (C and D) follicles in mouse ovarian tissue that received doses of 2, 5, and 10 mg/kg of WT1, WT2 and WTA. ^A,B^indicate differences in the comparison among CTR and 5 mg/kg of WT1, WT2, and WTA; ^a,b^indicate differences among CTR and 10 mg/kg of WT1, WT2, and WTA.

In Experiment I, a reduction (P < 0.05) in the percentage of primordial follicles and an increase (P < 0.05) in developing follicles were observed in the WT2 group compared to the CTR (Figure 2A and 2C). However, in experiment II, the percentage of primordial follicles decreased (P < 0.05) concomitantly with an increase (P < 0.05) in developing follicles (Figure 2B and 2D) in all groups (WT1, WT2, and WTA) at both concentrations compared to CTR.

**Figure 3:**
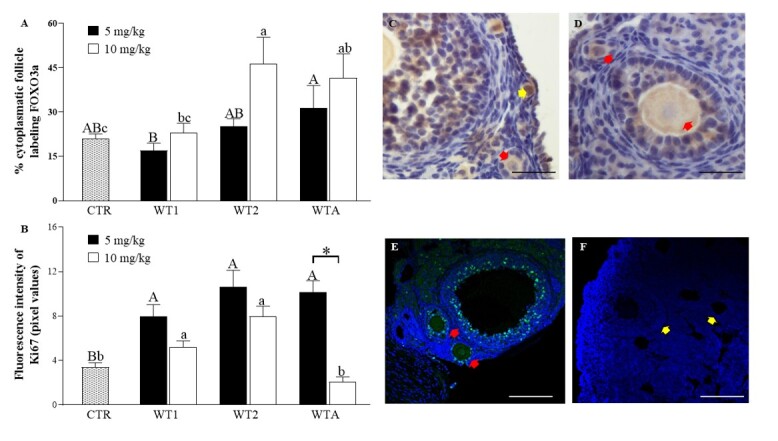
Immunohistochemical analysis of follicular activation (FOXO3a) (A) and granulosa cell proliferation (Ki67) (B). Representative images of FOXO3a (C and D) and Ki67 (E and F) immunostaining in ovarian follicles of mice that received doses of 5, and 10 mg/kg of WT1, WT2, and WTA. Yellow arrows indicate non-activated primordial follicles with nuclear staining of the oocyte (C), and red arrows indicate activated follicles with cytoplasmic staining marked by FOXO3a (C and D). Green points indicate granulosa cells (E) with presence of immunostaining for Ki67. The yellow arrows indicate absence of labeling (F). Scale bar = 50 μm. ^A,B^indicate differences in the comparison among CTR and 5 mg/kg of WT1, WT2, and WTA; ^a,b,c^indicate differences among CTR and 10 mg/kg of WT1, WT2, and WTA.

As shown in Figure 3A, in Experiment II, the expression of FOXO3a in the cytoplasm of oocytes present in ovarian follicles of animals treated with WT2 or WTA at 10 mg/Kg was higher (P < 0.05) than in the CTR. It should be noted that all follicles from all treated groups showed cytoplasmic staining for this protein, indicating signs of activation.

#### Granulosa cell proliferation in preantral follicles

In experiment II, a higher percentage (P < 0.05) of Ki67-positive cells was observed at a concentration of 5 mg/Kg in all groups compared to CTR (Figure 3B). At the concentration of 10 mg/Kg, immunostaining in the follicles of animals treated with WT1 or WT2 was higher (P < 0.05) than in the CTR. In the presence of WTA, immunostaining at 10 mg/Kg was lower than at 5 mg/Kg.

### Evaluation of ovarian degeneration indicators after WTI, WT2, and WTA administration

#### Presence of inflammation in the antral follicles

**Figure 4:**
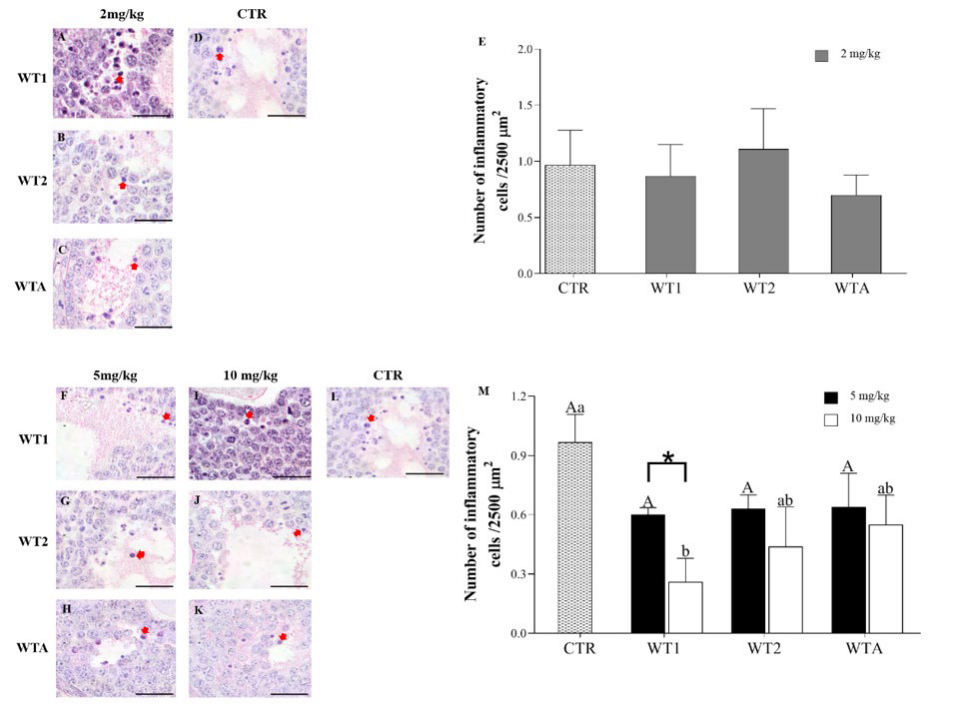
Representative images and average number (± s.e.m.) of inflammatory cells per 2500 µm2 in mice that received 2 mg/kg (A-D), 5 and 10 mg/kg (F-L) of WT1, WT2, and WTA. Analysis of inflammatory cells at a concentration of 2mg/Kg (E), 5 and 10 mg/Kg (M). ^A,B^indicates differences in the comparison among CTR and 5 mg/kg of WT1, WT2, and WTA. ^a,b^indicates differences among CTR and 10 mg/kg of WT1, WT2, and WTA. *Indicates differences between 5 and 10 mg/kg of the same withanolide. The arrows in the images of **Figure 5A**-F show inflammatory cells. Scale bar = 50 μm.

**Figure 5:**
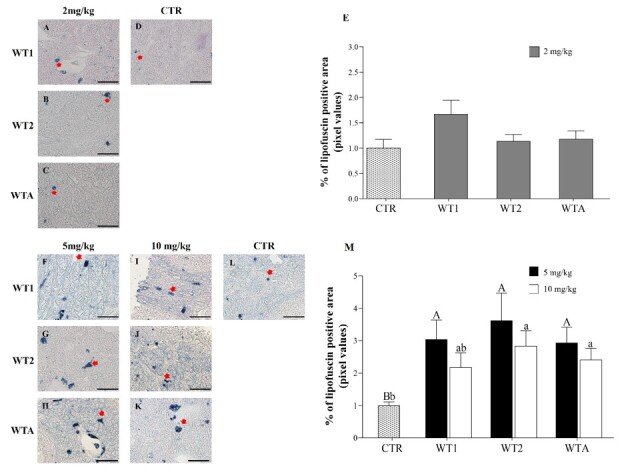
Representative images and average percentage (± s.e.m.) of Sudan Black staining per 2500 µm^2^ in mice receiving doses of 2 mg/kg (A-C), control (D), and 5 and 10 mg/kg (F-I) of WT1, WT2, and WTA. Analysis of senescent cells at concentrations of 2mg/Kg (E), 5, and 10 mg/Kg (M). ^A,B^indicate differences in the comparison between CTR and 5 mg/kg of WT1, WT2, and WTA; ^a,b^indicate differences between CTR and 10 mg/kg of WT1, WT2, and WTA. Scale bar = 50 μm.

The concentrations of 2 mg/Kg (experiment I; Figure 4E) and 5 mg/Kg (experiment II; Figure4M) of WT1, WT2, and WTA did not alter (P >0.05) the number of inflammatory cells present in antral follicles compared to CTR. However, at the concentration of 10 mg/Kg of WT1, a reduction (P < 0.05) in the number of inflammatory cells was observed compared to CTR and WT1 at the concentration of 5 mg/Kg.

#### Senescence in ovarian cells

Figure 5B and 5D show that there was no difference (P > 0.05) in the number of cells with lipofuscin granule accumulation in the ovaries of animals treated with 2 (Experiment I) and 5 mg/kg (Experiment II) of WT1, WT2, and WTA. However, in Experiment II, except for WT1 10 mg/kg, an increase (P > 0.05) in cells labeled by SBB was observed in the ovaries of animals treated with all drugs at both concentrations (5 and 10 mg/kg) compared to the CTR. Images in Figure 5A and 5C show cells with lipofuscin granule accumulation.

#### DNA damage in preantral follicles

##### Immunolocalization of γH2AX

**Figure 6:**
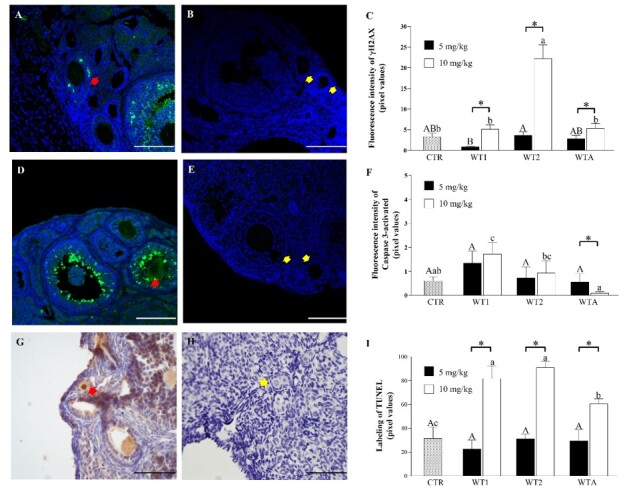
Immunohistochemical analysis of DNA damage by γH2AX (C), activated Caspase 3 (F) and TUNEL (I). Representative images of the immunostaining of γH2AX (A-B), activated Caspase 3 (D-E) and TUNEL (G-H) in the ovarian follicles of mice that received doses of 5 and 10 mg/kg of WT1, WT2, and WTA. The red arrows indicate follicles labeled for γH2AX (C), TUNEL (E). Activated caspase 3 (G) and yellow arrows indicate follicles not marked in the negative control for γH2AX (B), activated caspase 3 (E) and TUNEL (H). ^A,B^indicates differences in the comparison between CTR and 5 mg/kg of WT1, WT2, and WTA. ^a,b,c^indicates differences between CTR and 10 mg/kg of WT1, WT2, and WTA. μm *Indicates differences between 5 and 10 mg/kg of the same withanolide. Scale bar = 50 μm.

The data showed that the percentage of γH2AX-positive cells in preantral follicles present in the ovaries of animals treated with WT2 10 mg/kg was higher (P<0.05) than in the CTR (Figure 6C). Furthermore, it was observed that the percentage of labeled cells at a concentration of 10 mg/kg was higher (P<0.05) than at a concentration of 5 mg/kg in all withanolides.

##### Immunolocalization of activated Caspase 3

In Figure 6I, we observe that at the concentration of 5 mg/kg, the labeling pattern for activated Caspase 3 did not differ (P > 0.05) among CRT and the investigated drugs (WT1, WT2, and WTA). On the other hand, follicles from animals treated with WT1 10 mg/kg showed a higher labeling percentage (P<0.05) compared to the CTR. Interestingly, WTA 10 mg/kg showed less immunostaining (P<0.05) than at the 5 mg/kg concentration.

##### Immunolocalization of TUNEL

As shown in Figure 6F, at a concentration of 5 mg/kg, no significant differences (P > 0.05) in DNA fragmentation were observed among CRT and the WT1, WT2, and WTA drugs. However, at a concentration of 10 mg/kg, DNA fragmentation in the follicles of animals treated with all drugs (WT1, WT2, and WTA) was greater (P<0.05) than in the CTR. In the presence of WT1 or WT2, at a concentration of 10 mg/kg, DNA fragmentation was greater (P<0.05) than in the presence of WTA. Furthermore, DNA fragmentation at a concentration of 10 mg/kg was greater (P<0.05) than at 5 mg/kg in the presence of all the investigated drugs.

## Discussion

In general, chemotherapy drugs are used to damage or kill cells with a high proliferative capacity, a common characteristic of cancer cells, but unfortunately, they also induce damage to healthy cells in different tissues and organs, such as the ovary (Meirow et al., 2010; Zraik and Heß-Busch, 2021). Ovarian damage induced by chemotherapy mainly includes a reduction in follicular reserve, which can lead to infertility (Sonigo et al., 2019a; Mauri et al., 2020). Therefore, aiming to minimize these effects, several studies have investigated substances or metabolites of natural origin with anticancer properties (Rayan et al., 2017). In the present study, we demonstrate the side effects of withanolide derivatives (WT1, WT2, and WTA) on ovarian morphology and function.

Our results show that 2 mg/kg of WT1, WT2, and WTA did not present toxic effects on follicular morphology, ovarian function, and fertility in mice. Corroborating our results, Fong et al. (2012) reported that in an in vivo study, 2 mg/kg of WTA was also non-toxic to ovarian cancer cells, thus not preventing them from multiplying. On the other hand, at higher concentrations (5 and 10 mg/kg), a reduction in the population of preantral follicles considered morphologically normal was observed. Indeed, preantral follicles are highly sensitive to the action of chemotherapeutic agents, and depending on the concentration, they can cause a reduction in follicular reserve and premature aging during treatment (Bedoschi et al., 2016). Previous *in vivo* studies in humans also reported that WTA at higher concentrations was able to reduce tumor growth of pancreatic cells (Yu et al., 2010) and mammary cells (Stan et al., 2008) at doses of 6 mg/kg and 4 mg/kg, respectively. In our study, despite the population reduction, the follicular reserve was only partially depleted (~40%) at a concentration of 10 mg/Kg, not resulting in immediate ovarian follicular depletion. Cytotoxic effects of chemotherapeutic agents on the ovarian follicle population without causing total depletion have previously been observed in humans (Anderson et al., 2006) mice (Zhu et al., 2022) and rhesus monkeys (Ataya et al., 1995).

The data presented here demonstrate that the three withanolide derivatives at higher concentrations (5 and 10 mg/kg) were able to recruit dormant primordial follicles, activating them massively. It is known that the excessive recruitment of quiescent follicles, followed by growth stimulation and consequent reduction of the follicular reserve, known as superactivation or “burn out,” is one of the negative effects of chemotherapy (Nguyen et al., 2019; Kashi et al., 2023). In order to investigate the burnout phenomenon in our study, we analyzed cell proliferation and follicular activation through immunostaining for Ki67 and FOXO3a proteins, respectively. The data showed higher Ki67 labeling in ovaries of animals that received 5 mg/kg of WT1, WT2, and WTA, as well as 10 mg/kg of WT1 and WT2 compared to CRT. The Ki67 protein is expressed nuclearly in all phases of cell cycle (G1, S, G2 and mitosis) and absent in the G0 stage (Scholzen and Gerdes, 2000), being rarely expressed in primordial follicles due to their quiescent condition (Scalercio et al., 2015). Our findings indicated signs of increased labeling for FOXO3a in primordial follicles of animals treated with 10 mg/kg of WT2 and WTA compared to the control. FOXO3a is a transcription factor expressed in the nucleus of oocytes of primordial follicles, being exported to the cytoplasm as the follicles are activated and begin to grow (Hardy et al., 2018). Similar to other chemotherapy drugs (cisplatin: Kim et al., 2019; cyclophosphamide: Sonigo et al., 2019b; carboplatin: Ayhan et al., 2023), withanolide derivatives were able to overstimulate granulosa cell proliferation, leading to premature activation of primordial follicles and consequently an early reduction in follicle reserve. Although the withanolide derivatives stimulated follicular activation, the concentration of 5 mg/kg seems to be less harmful to the follicular reserve as the labeling for FOXO3a was similar to that observed in CTR.

We observed that, in general, withanolide derivatives at the three concentrations tested (2, 5, and 10 mg/kg) did not induce inflammation in follicular cells. Although unexpected, it is known that chemotherapy drugs induce cellular senescence, contributing to an increase in inflammatory cells (Schneider et al., 2017). This finding is consistent with the literature, as it has previously been reported that the withanolide family has an anti-inflammatory effect (Rasool and Varalakshmi, 2006). Furthermore, except for WT1 10 mg/kg, an increase in the accumulation of lipofuscin granules was observed in the presence of all drugs at both concentrations (5 and 10 mg/Kg) compared to CTR. Senescence is an indicator of ovarian degeneration that can occur in response to DNA damage triggered by cellular stressors, such as exposure to chemotherapeutic agents, often referred to as premature senescence, both *in vitro* and *in vivo* (te Poele et al., 2002).

Regarding the immunolabeling of proteins involved in the response to DNA damage, the expression of γH2AX significantly increased compared to control only in the group that received 10 mg/kg of WT2. The onset of DNA fragmentation during apoptosis initially induces the phosphorylation of histone H2AX at serine 139 (Rogakou et al., 1998; Svetlova et al., 2010). The immunolabeling of activated Caspase 3 was significantly higher in the presence of 10 mg/kg of WT1 compared to CTR. Caspase-3 is an important effector protease present in many cells as an inactive zymogen, namely procaspase-3. After activation, two subunits are generated which complex into a tetramer with a variety of cellular substrates and specifically induce activated DNase to cleave DNA, subsequently culminating in nuclear fragmentation (Shi, 2002). In addition, TUNEL labeling was significantly high at the concentration of 10 mg/kg, regardless of the withanolide derivative. The detection of ends of fragmented DNA strands represents a late event in the apoptosis process, which can be observed by TUNEL labeling (Loro et al., 1999; Gown and Willingham, 2002). Therefore, according to our findings, we hypothesize that the drugs used at the concentration of 5 mg/kg may not have caused sufficient DNA damage to sustain a detectable degree of γH2AX in the follicles, associated with the fact that no difference was observed in the labeling for activated Caspase-3 and TUNEL staining.

## Conclusion

In conclusion, the results of this study were very promising, as the three withanolide derivatives at the concentration of 2 mg/kg posed no risk to the population of preantral ovarian follicles or the reproductive function of mice, despite their cytotoxic effects on several cancer cell lines (data not shown). On the other hand, in the presence of higher concentrations, the risk is imminent, potentially leading to a burnout situation, as observed in the vast majority of chemotherapeutic agents widely used for cancer treatment, revealing that the cytotoxic effect of drugs on the ovary is concentration-dependent. Furthermore, we also found that at higher concentrations, such as 10 mg/kg, toxic effects are potentiated, causing DNA damage. We also suggest that in the absence of inflammation and evidence of cellular senescence, the DNA damage found may result from an apoptotic process.
